# Rationale, Design, and Baseline Characteristics of Participants in the Health@NUS mHealth Augmented Cohort Study Examining Student-to-Work Life Transition: Protocol for a Prospective Cohort Study

**DOI:** 10.2196/56749

**Published:** 2024-07-17

**Authors:** Xin Hui Chua, Sarah Martine Edney, Andre Matthias Müller, Nicholas A Petrunoff, Clare Whitton, Zoey Tay, Claire Marie Jie Lin Goh, Bozhi Chen, Su Hyun Park, Salome A Rebello, Alicia Low, Janelle Chia, Daphne Koek, Karen Cheong, Rob M van Dam, Falk Müller-Riemenschneider

**Affiliations:** 1 Saw Swee Hock School of Public Health National University of Singapore and National University Health System Singapore Singapore; 2 School of Medical and Health Sciences Edith Cowan University Joondalup Australia; 3 Health Promotion Board Singapore Government Singapore Singapore; 4 Departments of Exercise and Nutrition Sciences and Epidemiology Milken Institute of Public Health The George Washington University Washington, DC United States; 5 Yong Loo Lin School of Medicine National University of Singapore Singapore Singapore; 6 Digital Health Center Berlin Institute of Health Charité-Universitätsmedizin Berlin Berlin Germany

**Keywords:** wearable, wearables, movement behaviors, university students, mHealth, cohort study, data collection, well-being, young adults, health behaviors, physical health, Singapore, biometric assessment, questionnaire, Fitbit, smartwatch, smartphone app, app, application, sleep, dietary data, diet, dietary, psychological distress, distress, mobile phone

## Abstract

**Background:**

Integration of mobile health data collection methods into cohort studies enables the collection of intensive longitudinal information, which gives deeper insights into individuals’ health and lifestyle behavioral patterns over time, as compared to traditional cohort methods with less frequent data collection. These findings can then fill the gaps that remain in understanding how various lifestyle behaviors interact as students graduate from university and seek employment (student-to-work life transition), where the inability to adapt quickly to a changing environment greatly affects the mental well-being of young adults.

**Objective:**

This paper aims to provide an overview of the study methodology and baseline characteristics of participants in Health@NUS, a longitudinal study leveraging mobile health to examine the trajectories of health behaviors, physical health, and well-being, and their diverse determinants, for young adults during the student-to-work life transition.

**Methods:**

University students were recruited between August 2020 and June 2022 in Singapore. Participants would complete biometric assessments and questionnaires at 3 time points (baseline, 12-, and 24-month follow-up visits) and use a Fitbit smartwatch and smartphone app to continuously collect physical activity, sedentary behavior, sleep, and dietary data over the 2 years. Additionally, up to 12 two-week-long bursts of app-based ecological momentary surveys capturing lifestyle behaviors and well-being would be sent out among the 3 time points.

**Results:**

Interested participants (n=1556) were screened for eligibility, and 776 participants were enrolled in the study between August 2020 and June 2022. Participants were mostly female (441/776, 56.8%), of Chinese ethnicity (741/776, 92%), undergraduate students (759/776, 97.8%), and had a mean BMI of 21.9 (SD 3.3) kg/m^2^, and a mean age of 22.7 (SD 1.7) years. A substantial proportion were overweight (202/776, 26.1%) or obese (42/776, 5.4%), had indicated poor mental well-being (World Health Organization-5 Well-Being Index ≤50; 291/776, 37.7%), or were at higher risk for psychological distress (Kessler Psychological Distress Scale ≥13; 109/776, 14.1%).

**Conclusions:**

The findings from this study will provide detailed insights into the determinants and trajectories of health behaviors, health, and well-being during the student-to-work life transition experienced by young adults.

**Trial Registration:**

ClinicalTrials.gov NCT05154227; https://clinicaltrials.gov/study/NCT05154227

**International Registered Report Identifier (IRRID):**

DERR1-10.2196/56749

## Introduction

Noncommunicable diseases, such as cardiovascular diseases and type 2 diabetes, accounted for 74% of deaths globally [1] and contributed to 80% of Singapore’s total disease burden in 2019 [2], as leading causes of mortality [3,4]. The prevalence of people with chronic mental health conditions (eg, mood disorders and anxiety disorders) is also rising [5,6]. During the COVID-19 pandemic, 1 in 3 Singaporean youths aged 11 to 18 years reported symptoms associated with poorer mental health [7], and adults aged 18 to 34 years reported the highest proportion of mental health conditions [8]. Lifelong mental health conditions often begin in adolescence [9,10], emphasizing the importance of supporting the mental health of young adults [11].

The transitionary period from university to working life brings about numerous changes for young adults, such as increased work and financial responsibilities, alongside psychological developments as they adapt to new stressors [12-14]. Changes during the transitionary period are reported to have psychological impacts such as increasing stress levels or worsening mental health in young adults [15-17]. In response, some young adults may engage in health-promoting lifestyle behaviors, such as exercise [18] and healthy eating [19], to manage their stress, which may, in turn, improve their psychological, physical, and mental well-being [20]. In contrast, some young adults may engage in health-compromising behaviors, such as poor-quality diet, lower physical activity, or increased screen time [21-25], to manage their stress. Engaging in health-promoting behaviors (eg, healthy eating, exercise, and having adequate sleep) and refraining from risky behaviors (eg, binge drinking and smoking) is essential for preventing physical and mental illness in later life [26]. Additionally, health behaviors are often shaped in early adulthood and can persist throughout later life [11,23].

Despite the knowledge that health behaviors adopted in adolescence persist throughout adult life and influence health and well-being, gaps remain in understanding how environmental factors (eg, physical and social environment) influence changes in health and lifestyle behaviors during adolescence and early adulthood [27]. The complex and dynamic interplay between health behaviors often result in a synergistic effect on well-being [22]. For example, university students with poor mental well-being were more likely to consume sugar-sweetened beverages, consume alcohol, and smoke [28], and subsequentially are at an increased risk of chronic diseases in later adulthood [28,29]. As such, a rigorous understanding of health behaviors, their determinants, the interplay between multiple health behaviors, and the trajectories of health behaviors over time would deepen our understanding of their impact on the health and well-being of young adults [30]. Much of our current understanding comes from longitudinal studies using conventional data collection methods (eg, surveys, interviews, and telephone calls) that are vulnerable to recall biases and do not capture health behaviors, and the complex and dynamic interactions between them, in real-time, real-life settings [31-33].

Integration of mobile health (mHealth) data collection methods (eg, smartphones, wearable devices, and accelerometers) into cohort studies may allow the convenient collection of richer and more ecologically valid data [34], enabling deeper insights into individuals’ health and lifestyle behavioral patterns over time. mHealth allows continuous data collection throughout the day [33], capturing contextual information on health behaviors, as these occur in real time [35,36].

This paper aims to provide an overview of the study methodology and baseline characteristics of participants in Health@NUS, a longitudinal study leveraging mHealth to examine the trajectories in health behaviors, body weight, well-being, and their diverse determinants, for young adults during the student-to-work life transition.

## Methods

### Study Design and Setting

Health@NUS is a prospective cohort study conducted by researchers at the National University of Singapore (NUS) in partnership with the Singapore Government’s Health Promotion Board (HPB). Reporting of this study protocol follows the STROBE (Strengthening the Reporting of Observational Studies in Epidemiology) statement [37].

Health@NUS follows participants longitudinally over a 2-year period during university and transition to early working life. The study uses mHealth data collection methods, including a Fitbit smartwatch and the Health Insights SG (hiSG) mobile app developed by HPB, which collect data continuously over the 2-year period. Objective biometric measurements and questionnaires are collected at the 3 time points (baseline, 12-, and 24-month follow-up visits) with additional app-based ecological momentary surveys pushed out between the time points. Upon enrollment, participants also indicate whether they consent to be contacted for further follow-up assessments beyond the 2-year study period.

In addition to providing informed consent to join the study, participants were required to pay an SGD 20 (US $14.50) deposit to receive the Fitbit smartwatch (Versa Lite), which is refundable upon completion of the study. Participants were also required to setup a Direct Debit Authorization prior to approval to make a deduction from their bank account with HPB for SGD 248 (US $183) to recover the cost of the smartwatch should they lose it or withdraw from the study partway through.

### Participants and Recruitment

Participants were recruited between August 2020 and June 2022 via emails, internal student communication systems, and word-of-mouth. Data collection was expected to be completed by June 2024.

To be eligible, participants had to be (1) a full-time undergraduate or graduate university student, (2) a resident of Singapore, (3) aged between 18 and 26 years, and (4) own a smartphone with a Singapore registered mobile number and an iOS or Android operating system compatible with both the Fitbit app and hiSG mobile app. Students were excluded from participating if they (1) were pregnant at the point of recruitment, (2) traveled overseas for more than 6 weeks at a time, (3) were not willing to make an upfront deposit of SGD 20 (US $14), or (4) were not willing to set up a Direct Debit Authorization with HPB.

**Figure 1:**
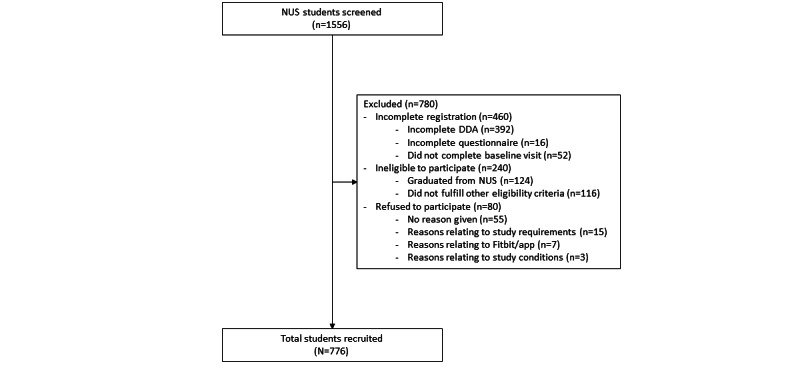
Participant flow diagram. DDA: Direct Debit Authorization; NUS: National University of Singapore.

Initially, between October 2020 and April 2021, only students who were between 1 and 3 semesters away from graduating were recruited. However, recruitment challenges were faced due to Singapore’s strict safety management measures during the COVID-19 pandemic; thus, this selection criterion was removed after April 2021. Students completed their digital registration, which included initial eligibility screening. Participants who were screened as eligible were invited to visit the study site to provide written informed consent and baseline biometric measurements (Figure 1). All study visits at baseline and 12- and 24-month follow-ups are conducted at the health screening site at NUS.

### Data Collection

**Figure 2:**
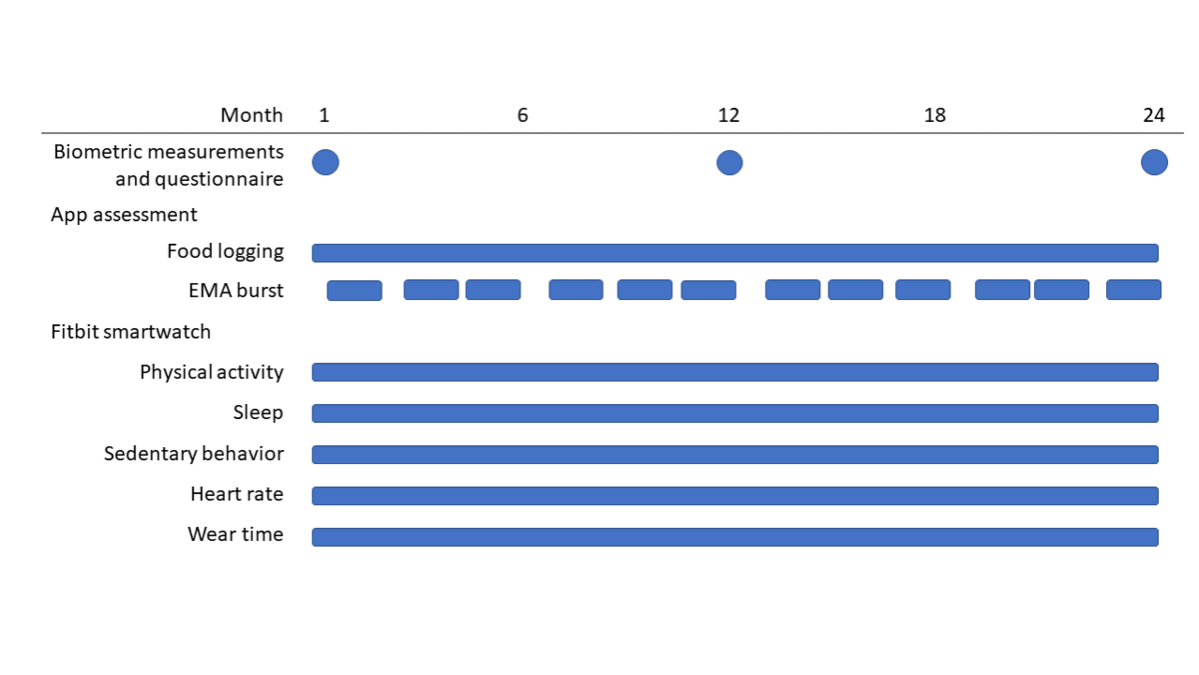
Timeline of assessments. Bars represent the continuous collection of data. Dots represent specific periods of data collection. EMA: ecological momentary assessment.

Biometric measurements, including height, weight, blood pressure, waist circumference, and resting heart rate, were conducted by trained staff using standardized protocols [38]. In addition, participants were issued a Fitbit smartwatch at baseline and were required to self-administer a web-based questionnaire on diet, physical activity, and other lifestyle factors and their underlying determinants at baseline, and the 12- and 24-month follow-up visits (Figure 2). Full details of the baseline questionnaire are presented in Table S1 in Multimedia Appendix 1 [38-79].

Repeated 2-week bursts of ecological momentary assessment (EMA) surveys were sent between the 3 main data collection time points (baseline, 12 months, and 24 months) via the hiSG app. Adapted from another ongoing study in Singapore [80], the EMA surveys contain questions relating to physical activity, diet, sleep, well-being, affect and physical feeling, behavioral cognitions (intentions and self-efficacy), cigarette smoking, alcohol consumption, and screen use (Table S2 and Figure S1 in Multimedia Appendix 1). Participants received an electronic voucher for completing the baseline assessments (SGD 5, US $3.50), each follow-up visit (worth SGD 50, US $36), and each of the EMA surveys was rewarded through Healthpoints (SGD 7, US $5).

### Fitbit Wear Time and hiSG App Logging Requirements

During the 2-year period, participants were asked to fulfill minimum data logging requirements relating to (1) wear time (awake and sleep) and (2) food logging (Table 1). Participants receive Healthpoints (150 Healthpoints=SGD 1, US $0.7) for completing the minimum study requirements across the 2-year study duration. The rewards would commensurate the level and duration of participation in the study and participants will receive milestone rewards of approximately SGD 80 (US $58) worth of Healthpoints (Figure S2A in Multimedia Appendix 1). Accumulated Healthpoints can be exchanged for electronic vouchers within the hiSG mobile app. Monthly reminders are sent via SMS text messages and emails to participants who do not fulfill the minimum logging requirements. Additionally, study participants are allowed to keep their Fitbit smartwatch at the end of the 2-year study period.

**Table 1:** Summary of minimum data logging requirements for waking wear time, sleep time, and food logging.

Activity and frequency			Minimum duration
**Wearing a smartwatch during waking hours**			
	Monthly	At least 8 hours a day, for at least 5 weekdays and 2 weekend days	
**Wearing a smartwatch to sleep**			
	Monthly	At least 1 weekday (Sunday to Thursday nights) and 1 weekend (Friday to Saturday nights)	
**Food diary: quarterly (timeline) according to the cycles**			
	1 (January 1-March 31)	All food and beverages consumed at least on 2 weekdays and 1 weekend day	
	2 (April 1-June 30)	All food and beverages consumed at least on 2 weekdays and 1 weekend day	
	3 (July 1-September 30)	All food and beverages consumed at least on 2 weekdays and 1 weekend day	
	4 (October 1-December 31)	All food and beverages consumed at least on 2 weekdays and 1 weekend day	

### Ethical Considerations

This study was conducted according to the guidelines laid down in the Declaration of Helsinki, and all procedures involving research study participants were approved by the National Health Care Group Domain Specific Review Board (Ref: 2019/00285, Singapore) and registered at ClinicalTrials.gov (NCT05154227). Written informed consent to participate in the study was obtained from all participants prior to data collection. Participants were offered SGD 120 (US $87) worth of Healthpoints for fulfilling the minimum data logging requirements, and up to SGD 425 (US $312) worth of Healthpoints were offered for additional wear time, sleep time, food logging, and survey data provided.

### Data Sources or Measurements or Outcomes

#### Web-Based Questionnaires

Table S1 in Multimedia Appendix 1 summarizes the variables collected from the self-administered questionnaires administered at 3 time points (baseline, 12-, and 24-month follow-up visits). The questionnaire collects sociodemographic information and assesses lifestyle behaviors and related factors. The questionnaire is administered via REDCap (Research Electronic Data Capture; Vanderbilt University) electronic data tools hosted at the NUS [81,82].

The collected sociodemographic data include age, ethnicity, sex, marital status, housing type, household income, current enrolled course of study, and year of study. Physical activity including time spent in different domains (active transport, occupational, leisure, and household) and sedentary behavior (including passive transport) were assessed using the modified version from the Singapore Prospective Study Program [38-40] validated for the local context. Screen time use across different devices was assessed using a modified version of the Adult Sedentary Behavior Questionnaire [41,42]. Items from the Pittsburgh Sleep Quality Index [43] assess sleep quality and quantity [43]. Dietary information comprises daily intake of key food groups (eg, fruits, vegetables, and whole grains) [44] assessed using standard serving sizes for each food item [83], alcohol consumption [38,40], dietary practices [44,45] (eg, type of fat or oil used for cooking, and type of milk or sweeteners consumed), and habitual eating behaviors [45-47].

Potential determinants of movement behaviors and diet are also assessed including cognitive determinants (eg, behavioral perceptions [48,49], self-efficacy [48,50,51], attitudes [51], intentions [52], plans [52], knowledge, personality [9,53], self-control [54], habit strength [55], and mental health and wellness [56-58]) and social determinants [48,51,59]. Assessment of the physical environment was adapted via the Physical Activity Neighborhood Environment Scale [60,61] and Food Environment Assessment in Singapore Tool. Mental health and wellness were assessed using the WHO (World Health Organization)-5 Well-Being Index (WHO-5) [58] and Kessler Psychological Distress Scale (K6) [56,57]. WHO-5 assesses positive well-being, where a cutoff score of less than or equal to 50 indicates reduced well-being [58]. K6 assesses psychological distress, where a cutoff score of greater than or equal to 13 indicates increased mental distress [56,57]. Questions on important events, including COVID-19 and the transition to working life, were added to the 12- and 24-month follow-up questionnaire.

#### Biometric Measurements

Height was measured using a portable stadiometer (Seca 213) without footwear and head positioned on the “Frankfurt” plane. The stadiometer headpiece was lowered and pressed firmly against the crown of the participant’s head. Height in meters was recorded to 3 decimal places. Weight was measured using an electronic scale (Seca 703) once participants removed all objects from their pockets, and recorded them to 2 decimal places in kilograms. According to the BMI classifications for the Asian population [4,84], overweight is defined as having a BMI between 23.0 and 27.4 kg/m^2^, while obesity is defined as having a BMI equal to or greater than 27.5 kg/m^2^. Waist circumference was measured using a standard measuring tape at the midpoint between the last rib and iliac crest during light expiration and recorded in centimeters to 1 decimal place.

Resting heart rate and blood pressure measurements were both taken twice using the Dinamap machine (Dinamap Carescape V100, General Electric), and the average of the readings was calculated. Participants were instructed to sit on a chair with their feet flat on the ground and their arms resting on a firm surface. The measurement was repeated 5 to 15 seconds after the cuff was fully deflated. Blood pressure was measured on the left arm unless contraindicated for blood pressure measurements.

#### Fitbit Smartwatch

At the baseline visit, participants set up their Fitbit smartwatch (Versa Lite) and their hiSG mobile app account. The Fitbit smartwatch passively collects data on physical activity such as step count, time spent active, calories burnt, heart rate, sedentary time, type of physical activity performed, and sleep. Participants were allowed to configure the Fitbit app for use on either their dominant or nondominant wrist. The data collected by the smartwatch are then synced with the Fitbit app, and subsequently transferred into the hiSG mobile app.

#### Smartphone-Based Assessments

The hiSG mobile app collects information on lifestyle behaviors, well-being, and their determinants through EMA surveys and dietary information through a food and beverage diary. hiSG monitors whether participants meet the minimum wear time and food logging study requirements, and participants can track whether these requirements are fulfilled within the app (“Progress,” Figure S2A in Multimedia Appendix 1). The “Healthpoints” tab of hiSG enables participants to view their accumulated Healthpoints and to exchange them for electronic vouchers (Figure S2B, Multimedia Appendix 1). Participants self-report their food and beverage intake via one of two methods: (1) text search for the food item consumed or (2) taking a photograph of the food item in the mobile app. The app then provides search results from which participants are required to select the closest matching food item from a local Singapore food database of items. Participants also indicate the portion size in household measures (such as cups or bowls) or grams and the approximate time that they ate the food by selecting one of the following time windows: midnight to 5 AM, 5 AM to 11 AM, 11 AM to 2 PM, 2 PM to 5 PM, 5 PM to 9 PM, or 9 PM to midnight. Figure S2C in Multimedia Appendix 1 shows an example of food and drink logging completed through the text search using the example of “kaya and butter toast” (a popular food item in Singapore). After each food item is logged, participants can click on the “see daily summary” tab to view a summary of foods and beverages logged for the day.

The 2-week bursts of EMA surveys commenced from April 2021 onward. Participants receive multiple bursts (lasting 1-2 weeks each) of EMA surveys each year, covering different phases of the academic calendar (instructional, examination, and vacation). Participants receive text messages and 2 in-app notification reminders to encourage them to respond to the EMA surveys. EMA surveys are spaced throughout the day, and participants have 45 minutes to answer each survey. The details of the EMA question constructs, response options, item source, and frequency of questions can be found in Figure S1 and Table S2 in Multimedia Appendix 1. Presented EMA content will be used in bursts 1 through 8 to cover the first year of follow-up. Subsequent EMA bursts will be adapted to cover a wider range of topics.

### Sample Size and Statistical Analysis

The study initially aimed to recruit 1300 students to account for attrition over the 2-year follow-up period and to achieve a target sample size of 1000. However, due to the challenges associated with recruitment during the COVID-19 pandemic, the original recruitment target was not met and 776 participants were recruited.

The sample size of 776 allows the detection of a small exposure effect (*f*^2^=0.02) on a continuous outcome when performing a multiple linear regression that accounts for 9 confounders at a significance level of 5% with more than 80% power [85,86]. The sample size of 776 also allows the detection of an odds ratio of 1.85 when performing a multiple logistic regression where 25% of the binary exposure’s variability is explained by the other predictors in the model, the probability of an outcome is between 0.2 and 0.7 among the unexposed, and the prevalence of being exposed is between 0.3 and 0.7 at a significance level of 5% with power at 80% or more [87]. The sample size (N=776) is larger than the number of participants recruited in previous EMA studies on youth [88-90].

Demographic data were analyzed descriptively. As the health and lifestyle behavior of university students are influenced by gender [91-93], comparisons of means between male and female participants were made via 2-tailed *t* test, and comparisons of medians between males and female participants were made via the Mann-Whitney *U* test. For categorical variables, differences between males and female participants were assessed by the chi-square test or the Fisher exact test for variables with small cell counts.

## Results

### Participant Flow

Interested participants (n=1556) were screened for eligibility prior to joining the study. We excluded 780 participants due to incomplete registration forms (n=460), ineligibility (n=240), and refusal to participate (n=80), as shown in Figure 2. A total of 776 participants were enrolled in the study.

### Participant Demographics

Of the 776 participants, 4 participants had incomplete baseline questionnaire data. The mean age of participants is 22.70 (SD 1.7) years. Participants are mostly female (441/776, 56.8%), Chinese ethnicity (714/776, 92%), undergraduate students (759/776, 97.8%), and not married (773/776, 99.6%). Half of the students (403/776, 51.9%) were in year 4 or year 5 of their undergraduate degree and were expected to transition into their working life during the study period. More details on participant demographics are presented in Table 2.

Table 3 shows anthropometric and lifestyle characteristics stratified by sex. Participants had a mean BMI of 21.9 (SD 3.3) kg/m^2^. Based on Asian classifications for BMI [84], 26.1% (202/773) were classified as being overweight (BMI ≥23 and <27.5 kg/m^2^) and 5.43% (42/773) were classified as being obese (BMI≥27.5 kg/m^2^). Participants had a mean systolic blood pressure of 113.7 (SD 11.42) mm Hg and a mean diastolic blood pressure of 64.76 (SD 7.94) mm Hg.

Most participants were nonsmokers (746/772, 96.6%), and more male participants (16/334, 4%) reported as being current or ex-smoker as compared to female participants (4/438, 1%). For dietary intake, the median intake of standard servings per day is reported in Table 3. Participants consumed 2.86 (IQR 1.71-4.64) servings of fruits and vegetables per day. Furthermore, participants reported consuming 1.53 (IQR 0.89-2.75) servings of refined grains as compared to 0.39 (IQR 0.08-1.00) servings of whole grains per day. For seafood, red meat, and poultry, participants consumed 0.71 (IQR 0.36-1.00) servings of poultry, 0.44 (IQR 0.23-0.86) servings of red meat, and 0.37 (IQR 0.20-0.64) servings of seafood per day. Only a small percentage of participants reported binge drinking (54/759, 7%).

For movement behaviors, participants engaged in an average of 0.78 (SD 0.76) hours per day in moderate to vigorous physical activity (MVPA), 10.87 (SD 3.60) hours of sedentary time, 13.39 (SD 4.22) hours of screen time, and 6.84 (SD 1.08) hours of sleep. For mental well-being, 37.7% (291/772) of the participants had poor mental well-being (WHO-5 score ≤50), and 14.1% (109/772) of the participants were at higher risk for psychological distress (K6≥13).

Significant differences between male and female participants (*P*<.05) were observed for all anthropometric measures (eg, height, weight, BMI, blood pressure, waist circumference, and average resting heart rate). Female participants were more likely to be underweight and had a higher resting heart rate, while male participants were more likely to be overweight. Significant differences between males and female participants were observed only in a few lifestyle behaviors such as consumption of refined grains and red meat, time spent in MVPA, and mental well-being and psychological distress (Table 3). Male participants consumed more refined grains and red meat and spent more time engaging in MVPA than female participants. A larger proportion of females reported poor mental well-being and psychological distress, as compared to male participants.

**Table 2:** Demographics of participants at baseline.

Characteristics			Total (N=776)		Male (n=335)		Female (n=441)
Age (years), mean (SD)			22.70 (1.7)		23.58 (1.6)		22.20 (1.4)
**Ethnicity, n (%)**							
	Chinese	714 (92)		305 (91)		409 (92.7)	
	Malay	20 (2.6)		10 (3)		10 (2.3)	
	Indian	24 (3.1)		13 (3.9)		11 (2.5)	
	Others	18 (2.3)		7 (2.1)		11 (2.5)	
**Student type, n (%)**							
	Undergraduate	759 (97.8)		328 (97.9)		431 (97.7)	
	Postgraduate	17 (2.2)		7 (2.1)		10 (2.3)	
**Faculty of study, n (%)**							
	Science	204 (26.3)		69 (20.6)		135 (30.6)	
	Medicine, nursing, and dentistry	149 (19.2)		53 (6.8)		96 (12.4)	
	Engineering	130 (16.8)		93 (27.8)		37 (8.4)	
	Arts and social sciences	108 (13.9)		32 (9.6)		76 (17.2)	
	Business and accountancy	73 (9.4)		34 (10.2)		39 (8.8)	
	Design and environment	55 (7.1)		21 (6.3)		34 (7.7)	
	Computing	37 (4.8)		24 (7.2)		13 (3)	
	Others	20 (2.6)		9 (2.7)		11 (1.4)	
**Level of study, n (%)**							
	Year 1	134 (17.3)		70 (20.9)		64 (14.5)	
	Year 2	64 (8.1)		26 (7.8)		38 (8.6)	
	Year 3	175 (22.5)		68 (20.3)		107 (24.3)	
	Year 4	347 (44.7)		150 (44.8)		197 (44.7)	
	Year 5	56 (7.2)		21 (6.3)		35 (7.9)	
**Residence, n (%)^b^**							
	On campus	112 (14.5)		50 (15)		62 (15)	
	Off campus	661 (85.5)		284 (85)		377 (85.9)	
**Marital status, n (%)**							
	Never married	773 (99.6)		332 (99.4)		440 (99.8)	
	Currently married	3 (0.4)		2 (0.6)		1 (0.2)	
**Monthly family^a^ income (SGD, 1 SGD=US $1.38), n (%)^b^**							
	<$2000	69 (8.9)		36 (10.8)		33 (7.5)	
	$2000-$3999	112 (14.5)		44 (13.1)		68 (15.4)	
	$4000-$5999	108 (14)		50 (14.9)		58 (13.2)	
	$6000-$9999	122 (15.8)		68 (20.3)		54 (12.2)	
	>$10,000	156 (20.2)		60 (17.9)		96 (21.8)	
	Refuse to answer or do not know	206 (27.8)		56 (16.7)		130 (29.5)	

^a^Monthly family income refers to the income received by all family members (eg, parents).

^b^Missings residence (n=3), and monthly family income (n=3).

**Table 3:** Anthropometric measures and lifestyle profiles of participants at baseline (n=773).

Characteristics					Total			Male (n=335)			Female (n=441)			*P* value	
**Anthropometric measures (n=773^a^), mean (SD)**															<.001
	Height (m)			1.66 (0.09)			1.73 (0.06)			1.60 (0.06)					
	Weight (kg)			60.67 (11.91)			68.66 (10.44)			54.63 (9.07)					
	BMI (kg/m^2^)			21.91 (3.29)			22.88 (3.26)			21.20 (3.11)					
**BMI category (kg/m^2^; n=773^a^), n (%)**															<.001
	Underweight (<18.5)			91 (11.8)			15 (4.5)			76 (17.3)					
	Normal (18.5-<23)			438 (56.7)			172 (51.7)			266 (60. 5)					
	Overweight (23-<27.5)			202 (26.1)			121 (36.3)			81 (18.4)					
	Obese (≥27.5)			42 (5.4)			25 (7.5)			17 (3.9)					
Waist circumference (cm; n=773^a^), mean (SD)					73.09 (8.6)			77.46 (8)			69.78 (7.4)			<.001	
**Blood pressure (mm Hg; n=767^a^), mean (SD)**															<.001
	Systolic			113.68 (11.42)			120.76 (9.61)			108.33 (9.65)					
	Diastolic			64.76 (7.94)			67.17 (7.50)			62.93 (7.78)					
	Average resting heart rate			71.59 (11.55)			69.28 (11.55)			73.35 (11.56)					
**Lifestyle behaviors**															
	**Cigarette smoking status (n=772^a^), n (%)**														.003
		Never smoker	746 (96.6)			313 (93.7)			433 (98.9)						
		Ex-smoker	9 (1.2)			7 (2.1)			2 (0.5)						
		Current smoker	11 (1.4)			9 (2.7)			2 (0.5)						
		Refuse to answer	6 (0.8)			5 (1.5)			1 (0.2)						
	**Dietary intake (servings per day; n=773^a^), median (IQR)**														
		Refined grains	1.53 (0.89-2.75)			1.87 (1.16-2.97)			1.38 (0.80-2.57)			<.001			
		Whole grains	0.39 (0.08-1.00)			0.42 (0.08-1.00)			0.39 (0.08-0.91)			.44			
		Seafood	0.37 (0.20-0.64)			0.43 (0.21-0.80)			0.32 (0.19-0.58)			.003			
		Red meat	0.44 (0.23-0.86)			0.71 (0.36-1.07)			0.44 (0.17-0.71)			<.001			
		Poultry	0.71 (0.36-1.00)			0.71 (0.36-1.00)			0.71 (0.36-0.71)			<.001			
		Fruits and vegetables	2.86 (1.71-4.64)			3.02 (1.79-4.64)			2.72 (1.68-4.61)			.25			
		Sugar-sweetened beverage	0.08 (0.03-0.14)			0.08 (0.03-0.14)			0.08 (0.03-0.14)			.89			
Binge drinking^b^ (n=759^a^), n (%)					54 (7.1)			27 (8.3)			27 (6.2)			.26	
**Movement behavior, mean (SD)**															
	Time spent in MVPA^c^ (hours/day; n=773^a^)			0.78 (0.76)			0.96 (0.78)			0.65 (0.71)			<.001		
	Total sedentary time (hours/day; n=772^a^)			10.87 (3.60)			10.82 (3.63)			10.91 (3.58)			.73		
	Total screen time (hours/day; n=772^a^)			13.39 (4.22)			13.40 (4.17)			13.39 (4.27)			.89		
	Sleep duration (hours; n=771^a^)			6.84 (1.08)			6.78 (0.99)			6.89 (1.13)			.15		
**Mental well-being (n=772^a^), n (%)**															
	K6^d^ score ≥13			109 (14.1)			35 (10.5)			74 (16.9)			.02		
	WHO-5^e^ score ≤50			291 (37.7)			110 (32.9)			181 (41.3)			.02		

^a^Incomplete baseline questionnaire data.

^b^Binge drinking is defined as consuming 5 or more servings of alcohol for males, or 4 or more servings of alcohol for females in a single drinking session in the past month.

^c^MVPA: moderate to vigorous physical activity.

^d^K6: Kessler Psychological Distress Scale.

^e^WHO-5: World Health Organization–5 Well-Being Index.

## Discussion

### Principal Findings

Health@NUS is the first longitudinal study of its kind to explore the interrelationships and associations among health behaviors, health, and well-being of young adults as they transition from university and into their working lives. In addition to traditional assessments used in cohort studies (self-report surveys and physical examination), Health@NUS uses mHealth technologies for continuous and repeated assessments of health behaviors and related contextual information at a level of granularity that has not previously been possible [36]. These data will provide important insights into the determinants and trajectories of health behaviors, health, well-being and related contextual information, and the dynamic and synergistic interrelationships between them.

With an increasing number of young adults graduating from universities in Singapore [94] and across the world, more young adults are subjected to this transitionary period between university and working life [6]. Understanding young adults’ health behaviors, health, and well-being during this vulnerable period in their lives, how they change, and what influences these changes has important implications for the promotion of population health. The early results from Health@NUS baseline assessments provide insights into indicators of physical health (eg, BMI, waist circumference, and blood pressure) among university students in Singapore. For instance, participants of Health@NUS reported an average BMI of 21.91 kg/m^2^, and the prevalence of obesity (5.43%) was substantially lower than among all Singapore residents aged 18 to 29 years (13.1%) [4]. This may be in part due to the low age of the study population, even though most weight gain occurs between the ages of 18 and 29 years in Singapore [95]. The proportion of underweight female participants (n=91, 17.3%) was greater than Singapore residents (10%) [4], which is an underrecognized public health problem prevalent among young female participants [96]. Further research is warranted to investigate this issue. In addition, the average waist circumference for both male (77.46 cm) and female (69.78 cm) participants was lower than the cutoffs for abdominal obesity in Asian populations [4,97]. Moreover, the prevalence of smoking (11/772, 1.4%) and binge drinking (54/759, 7.1%) among participants of Health@NUS was very low compared to Singapore residents aged 18 to 29 years who reported a prevalence of smoking and binge drinking of 8.3% and 15.6%, respectively [98]. The prevalence of smoking and binge drinking in this study population was also low in comparison to other studies among university students in Singapore and globally [99-102].

In contrast, baseline data on movement behaviors and diet present a less favorable outlook of the student population. While physical activity levels and sleep duration appear low, time spent being sedentary and using screen devices is high. This is in contrast to the physical activity levels observed among Singapore residents aged 18 to 29 years, who were reported to be most likely to engage in regular exercise [4,98]. Compared to Singapore residents aged 18 to 29 years (76.3%), our findings suggest that fewer students (n=555, 72.7%) met the minimum recommendations of at least 150 minutes per week of MVPA as defined by the World Health Organization guidelines [4,103,104]. This was particularly obvious among female students, for whom the proportion meeting recommendations was markedly lower than among their male counterparts. Furthermore, time spent in sedentary behavior among Health@NUS participants was greater than in other studies in Singapore, which reported an average of 5 to 6 hours per day [105,106]. Low levels of physical activity and large amounts of time spent in sedentary behavior by Health@NUS participants may in part be attributable to the fact that university students spent the longest time per week globally studying [107]. With regard to the consumption of whole grains, fruits, and vegetables, the situation also appears suboptimal compared to the “My Healthy Plate” dietary recommendations from the HPB for Singapore residents [108]. On average, students did not meet the recommended daily 5 to 7 servings of whole grains, and daily 4 servings of fruits and vegetables [109]. The daily consumption of sugar-sweetened beverages on the other hand was exceptionally low as compared to Singapore residents [110,111], where sugar-sweetened beverages accounted for 52% of dietary sugar intake [111]. These findings warrant further investigation based on Health@NUS’s extensive diet logging and EMA data to better understand dietary patterns of university students in Singapore.

The high prevalence of poor mental well-being (291/772, 37.7%) and psychological distress (109/772, 14.1%) observed in this study population of young adults is of great concern. Our findings from the WHO-5 scores suggest that university students in Singapore have poorer mental well-being as compared to other countries such as Germany [112], New Zealand [113], Hong Kong [114], the United Kingdom [115], and the United States [116]. According to national studies in Singapore, poor mental well-being is most prevalent in young adults aged 18 to 29 years residing in Singapore [4,8], and the proportion of young adults with poor mental well-being has been gradually rising in recent years [4,8,98]. In comparison to the Singapore Mental Health Study 2016, Singapore residents aged between 18 and 34 years reported the highest prevalence of lifetime- and 12-month mental disorders at 21.6% and 11.9%, respectively [8]. Furthermore, the prevalence of poor mental well-being in Singapore prior to and during the COVID-19 pandemic was found to be 8.4% and 8.7%, which is lower than reported in Health@NUS [117,118]. As most mental illnesses (eg, anxiety and depression) [6] emerge during adolescence or early adulthood [9,119,120], the delay in help-seeking by affected individuals could have long-lasting emotional and social impacts on their adulthood [119,121]. Greater levels of stress experienced by students in university increase their vulnerability toward mental health conditions (eg, depression), substance abuse (eg, alcohol abuse), and chronic health conditions (eg, obesity) [6] in later adulthood without proper coping mechanisms. As modifiable lifestyle behaviors (eg, physical activity, diet, and sleep) are associated with positive mental well-being [122], it is important for Health@NUS to explore the underlying determinants of students’ health behaviors during the transition period from university to working life. To this end, data collected from Health@NUS will be used to inform the design of future interventions to promote healthy behaviors and the health and well-being of students during these formative years and beyond. Given recent attention on the poor mental health and well-being of young people in Singapore [4,5,7,8,119,121], 1 of 4 priorities will also be to use the detailed data from Health@NUS to understand trajectories of well-being and psychological distress, as well as their related contextual factors over time, and to identify potential intervention strategies to promote well-being among the student population in Singapore.

Health@NUS will provide unprecedented information on health behaviors, health, and well-being, and related contextual information. We have recruited a large sample and followed these students for 2 years, over their time at university and as they transition into their postgraduation working life. Combining assessments used in traditional cohort studies (self-reported questionnaires and biometric assessments), as well as repeated and continuous data collection via smartwatches and smartphones will provide detailed longitudinal data. Importantly, much of the data collected required minimal inputs from participants (ie, data from the smartwatch and smartphone), and we captured these data in real time and as participants were going about their daily lives, which enhances the ecological validity of our findings. A potential limitation of presented results relates to the self-reported lifestyle behaviors (eg, dietary intake, cigarette smoking, alcohol consumption, and physical activity). Future analysis of data collected via smartwatches, food logging, and EMA will help to overcome this limitation to some extent. Additionally, there could be a possibility of selection bias as health-conscious and motivated students were more likely to join the study. Disruptions stemming from the COVID-19 pandemic might have affected the job opportunities of university students seeking employment, thus intensifying their stress levels during the challenging transition from study to work. This will have implications on the generalizability of our findings to the wider university student population in Singapore and emphasizes the need for further research in the post–COVID-19 era.

### Conclusions

To our knowledge, Health@NUS is a novel prospective cohort study that augments information collected from traditional questionnaires and on-site health screening, with mHealth technologies to capture intensive longitudinal data on individual characteristics, lifestyle behaviors, health, and well-being among young adults during their transition from university to working life in real life [34,35]. Health behaviors shaped in early adulthood often persist into adulthood [11,23] and can have negative implications for health and well-being. A deeper understanding of how health behaviors cluster or interact and of the influence of individual and ecological determinants is essential to developing effective health promotion strategies for young adults in Singapore. Furthermore, the findings from this study will provide insights into the mental health of students during the potentially stressful transition from university to work life.

## Appendix

Multimedia Appendix 1 Supplementary tables and figures.

## Abbreviations

EMA: ecological momentary assessment
hiSG: Health Insights SG
HPB: Health Promotion Board
K6: Kessler Psychological Distress Scale
mHealth: mobile health
MVPA: moderate to vigorous physical activity
NUS: National University of Singapore
REDCap: Research Electronic Data Capture
STROBE: Strengthening the Reporting of Observational studies in Epidemiology
WHO-5: World Health Organization–5 Well-Being Index

## Data Availability

The data sets used or analyzed during this study are available from the corresponding author on reasonable request.
