# Supplementary material for: Rationale, Design, and Baseline Characteristics of Participants in the Health@NUS mHealth Augmented Cohort Study Examining Student-to-Work Life Transition: Protocol for a Prospective Cohort Study
Source: JMIR Res Protoc. 2024 Jul 17;13:e56749. doi: 10.2196/56749 (PMC11292158; doi:10.2196/56749)
Supplement: Multimedia Appendix 1 [file resprot_v13i1e56749_app1.docx]

**Supplementary materials**

Supplementary Table S1. Baseline questionnaire – constructs, survey instruments, description, and assessment period of item

Supplementary Table S2. Ecological Momentary Assessment – constructs, survey instruments, and prompting schedule

Supplementary Figure S1. Tree plot showing the question branching logic of Ecological Momentary Assessment

Supplementary Figure S2. Screenshots of hiSG mobile application

1. Summary of minimum logging requirements for food logging, wear time and sleep time
2. Healthpoints tab for participants to claim and redeem their healthpoints for e-vouchers
3. Meal logging using the text search function of a food item using the example of “kaya and butter toast”

**Supplementary Table S1.** Baseline questionnaire – constructs, survey instruments, description, and assessment period of items

| **Construct** | **Instrument/ source** | **Description of items** | **Assessment period** | | |
| --- | --- | --- | --- | --- | --- |
|  |  |  | **Baseline visit** | **12-month follow up** | **24-month follow up** |
| **Movement behaviours** | | | | | |
| Physical activity | Singapore Prospective Study Program (SP2PAQ) [39] | 13-items assessing sedentary behaviour, transportation, occupational, leisure, and household activity domains of physical activity | ✓ | ✓ | ✓ |
| Passive transport | Modelled on SP2PAQ [39], used in local context [38, 40] | 2-items   1. In a typical week, how many days do you drive or ride motorized transport to get to and from places? 2. How much time would you spend sitting during motorized transport (e.g. car, bus or MRT) for travel on a day? | ✓ | ✓ | ✓ |
| Study-related sedentary behaviour | Modelled on SP2PAQ [39] | 2-items   1. On average, how many hours per weekday and weekend day do you spend sitting down, reclining or lying down while doing activities in your free time (e.g. reading for pleasure, eating or watching movies? 2. On average, how many hours per weekday and weekend day on study- or work-related activities (e.g. doing assignments, sitting down in lectures or meetings)? | ✓ | ✓ | ✓ |
| Screen time | Screen-time questionnaire [42] and modelled on domain-specific Adult Sedentary Behaviour Questionnaire (ASBQ) [41] | 6-items assessing the duration of use of different devices on weekday and weekend days for study/work purposes.   1. On average, how many hours per weekday do you spend watching movies/TV/DVDs for entertainment (not on a mobile device)? 2. On average, how many hours per weekend day do you spend watching movies/TV/DVDs for entertainment (not on a mobile device)? 3. On average, how many hours per weekday do you spend looking at a screen of a mobile device?    - How much of this time is for study/work purposes? 4. On average, how many hours per weekend day do you spend looking at a screen of a mobile device?    - How much of this time is for study/work purposes? 5. On average, how many hours per weekday do you spend looking at a computer/laptop?    - How much of this time is for study/work purposes? 6. On average, how many hours per weekend day do you spend looking at a computer/laptop?    - How much of this time is for study/work purposes? | ✓ | ✓ | ✓ |
| Sleep | Pittsburgh Sleep Quality Index (PSQI) [43] | 16-items assessing sleep quality and quantity | ✓ | ✓ | ✓ |
| Neighbourhood environment  (outdoors) | Physical Activity Neighbourhood Environment Scale (PANES) [60] | 5-items assessing how physical-activity friendly the outdoor environment is perceived   1. Many exercise facilities are on campus/around my workplace. 2. There are many public transport stops nearby. 3. There are sidewalks around campus/my workplace. 4. There are facilities on campus/around my workplace that allow cycling (special lanes, separate paths, shared use paths for pedestrians and bicycles). 5. Many sport and exercise courses that appeal to me are offered by the university/my workplace | ✓ | ✓ | ✓ |
| Indoor environment | Modelled on [61] | 2- items   1. How many pieces of sports and/or exercise equipment do you have at the place you live (e.g. weights, treadmill, rubber band)? 2. How many screen-based devices do you have access to at the place you live (e.g. computer, TV, game console)? | ✓ | ✓ | ✓ |
| **Food and eating** | | | | | |
| Diet intake | 37-item Food Frequency Questionnaire (FFQ) [44] | 37-items assessing the frequency of eating/drinking different foods – designed for use in local multi-ethnic Asian population [38, 40] | ✓ | ✓ | ✓ |
| Dietary practices | Food Frequency Questionnaire [45] designed for multi-ethnic Asian population, used in local cohort studies [38, 40] | 16-items on usual dietary practices related to where food is purchased (on campus, hawker centres, fast food restaurants, other restaurants), types of fat/oil used for cooking, salt/condiments added at the table, type of milk/sweetener consumed | ✓ | 🗶 | 🗶 |
| Habitual eating behaviours | 18-item Three Factor Eating Questionnaire [45-47] | 18-items assessing cognitive restraint, uncontrolled and emotional aspects of habitual eating behaviours | ✓ | 🗶 | 🗶 |
| Mindful eating | Used in local cohort studies [38, 40] | 1-item   1. How often do you eat mindfully? To eat mindfully would mean, paying attention to how hungry you are and taking the time to savour your food | ✓ | ✓ | ✓ |
| Eating patterns | Used in local cohort studies [38, 40] | 2-items   1. How many times per day do you eat? Include meals and snacks, but exclude all beverages 2. In a typical week, on how many days do you eat breakfast? | ✓ | ✓ | ✓ |
| Dietary restrictions | Modelled on Food Environment Assessment in Singapore Tool (FEAST) – perceived | 2-items   1. Do you currently follow a special diet or have any dietary restrictions? Note: this includes dietary restrictions due to religious/health/personal reasons. If yes, ask: 2. What type of special diet are you following? (Weight-loss diet, diet recommended for medical reasons (please specify), vegetarian/vegan, exclusion of pork/pork products, other (please specify) | ✓ | 🗶 | 🗶 |
| Binge drinking | Used in local cohort studies [38, 40] | 1-item  Did you have 4 (if female)/ 5 (if male) or more servings of alcohol at a single drinking session in the past month? | ✓ | ✓ | ✓ |
| Food environment |  | 2-items   1. I have access to cooking facilities where I live 2. Many healthy food options are on the campus/ around my workplace. | ✓ | ✓ | ✓ |
| **Psychology, Personality, and Mental Health** | | | | | |
| Attitudes | Modelled on [51] | 6-items   1. Being physically active is beneficial. 2. Being physically active is enjoyable. 3. Spending time being sedentary (ie, sitting, lying down, reclining during waking hours) is beneficial. 4. Spending time being sedentary (ie, sitting, lying down, reclining during waking hours) is enjoyable. 5. Eating healthily (eg, more fruits and vegetables, less sugar and meat) is beneficial. 6. Eating healthily (eg, more fruits and vegetables, less sugar and meat) is enjoyable. | ✓ | 🗶 | 🗶 |
| Behavioural perceptions | Modelled on [48, 49] | 7-items   1. I regularly walk 10,000 steps per day. 2. I regularly do 150 minutes of moderate-to-vigorous physical activity per week. 3. I regularly sit less than 8 hours per day. 4. I regularly interrupt long periods of sitting with breaks. | ✓ | 🗶 | 🗶 |
|  |  | 1. I regularly eat 2 vegetables and 2 fruits per day. 2. I regularly avoid sugary drinks/snacks. 3. I regularly choose low calorie foods. |  |  |  |
| Habit strength | Self-Report Behavioural Automaticity Index (SRBAI) [55] | 8-items assessing automaticity (control, awareness, efficiency) in relation to physical activity, sedentary behaviour, and eating healthily (total 24-items) | ✓ | 🗶 | 🗶 |
| Intentions | Modelled on [52] | 4-items  Over the next two months I intend to…   1. be more physically active. 2. reduce the time I spend being sedentary (eg, sitting) 3. eat more fruits and vegetables 4. reduce the amount of sugary drinks I consume | ✓ | 🗶 | 🗶 |
| Knowledge |  | 2-items   1. Being sedentary means…(spending a lot of time sitting or lying down, not being active enough, spending a lot of time sitting or lying down and not being active enough, don’t know) 2. Doing some physical activity can offset the negative effects of sitting too much. | ✓ | ✓ | ✓ |
| Personality | Ten Item Personality Inventory (TIPI) [53] | 10-item assessing the big five personality traits (agreeableness, conscientious, emotional stability, extraversion, openness to experience). | ✓ | 🗶 | 🗶 |
| Plans | Plans for physical activity modelled on [52], adapted for sedentary behaviour and healthy eating | 8-items  I have made plans…   1. about when, where and how I will do physical activity 2. what kind of physical activity I will do 3. what I will do if my initial physical activity plan cannot be implemented 4. when and where to reduce the time I spend being sedentary. 5. what activity I will do when I interrupt sedentary time 6. about when and where I will choose healthy food options 7. what healthy food options I will choose 8. what I will do itmy initial healthy eating plan cannot be implemented | ✓ | 🗶 | 🗶 |
| Psychological distress | Kessler 6 (K6) [56, 57] | 6-items assessing psychological distress | ✓ | ✓ | ✓ |
| Self-control | Brief Self-Control Scale (BSCS) [54] | 13-items assessing self-control | ✓ | 🗶 | 🗶 |
| Self-efficacy | Modelled on [48, 50, 51] | 7-items  It is up to me…   1. how much physical activity I do 2. how much time I spend being sedentary 3. what kind of food I eat   I am confident that I could…   1. be more physically active in the next month, if I wanted to 2. reduce my sedentary time in the next two months, if I wanted to 3. compare foods to select healthier packaged food options 4. compare foods to make healthier choices when I eat out | ✓ | 🗶 | 🗶 |
| Social norms | Modelled on [48, 51, 59] | 9-items assessing social norms in relation to physical activity, sedentary behaviour, and diet, asked in relation to the significant other, family, friends (total 27-items).  To what extent does your significant other:   1. Drink sugar-sweetened beverages 2. Eat fast food 3. Eat fruits and vegetables 4. Prepare meals at home 5. Encourage you to be physically active 6. Invite you to be physically active with them 7. Do physical activity with you 8. Encourage you to reduce the time you are sedentary | ✓ | ✓ | ✓ |
| Well-being | WHO Five Well-being Index (WHO-5) [58] | 5-items assessing current well-being | ✓ | ✓ | ✓ |
| **Medical information** | | | | | |
| Medical history | Used in local cohort studies [38, 40] | 3-items   1. Have you ever been diagnosed by a Western-trained doctor for a chronic medical condition? If yes, please specify. 2. Do you have an illness or condition that restricts your movement? 3. Do you have any medical condition that affects your diet? | ✓ | ✓ | ✓ |
| Women’s health | Used in local cohort studies [38, 40] | 1-item   1. Are you currently pregnant? | ✓ | ✓ | ✓ |
| Smoking status | Used in local cohort studies [38, 40] | 1-item   1. Which of the following best describes you? (daily smoker, occasional smoker, ex-smoker, non-smoker, refuse to answer). | ✓ | ✓ | ✓ |

**Supplementary Table S2**. Ecological Momentary Assessment – constructs and survey instruments

| **Construct** | **Question** | **Response options** | **Item source** |
| --- | --- | --- | --- |
| **Sleep** | | | |
| Sleep quality | How would you rate your overall sleep quality last night? | Likert scale   - 0=very bad, 6=very good | Adapted from Pittsburgh Sleep Quality Index [43] |
| Timing | What time did you go to bed last night? | Enter time |  |
| Time to fall asleep | How long did it take you to fall asleep last night? | - Few minutes - About half an hour - About 1 hour - More than 1 hour |  |
| **Affect & feelings** | | | |
| Stress | How stressed do you feel right now? | Likert scale   - 0=not at all, 6=very much | Adapted from EMA studies [62-65] |
| Affect | How happy do you feel right now? | Likert scale   - 0=not at all, 6=very much | Adapted from PANAS [66] and EMA study [67] |
| Fatigue | How tired do you feel right now? | Likert scale   - 0=not at all, 6=very much | Adapted from EMA studies [62, 63, 65] |
| Mental wellbeing | Over the last 2 weeks, I have felt cheerful and in good spirits | Likert scale   - 0=at no time, 5=all the time | Adapted from WHO Five Well-being Index (WHO-5) [58] |
|  | Over the last 2 weeks, I have felt calm and relaxed. | Likert scale   - 0=at no time, 5=all the time |  |
|  | Over the last 2 weeks, I have felt active and vigorous. | Likert scale   - 0=at no time, 5=all the time |  |
|  | Over the last 2 weeks, I woke up feeling fresh and rested | Likert scale   - 0=at no time, 5=all the time |  |
|  | Over the last 2 weeks, my daily life has been filled with things that interest me. | Likert scale   - 0=at no time, 5=all the time |  |

| **Construct** | **Question** | **Response options** | **Item source** |
| --- | --- | --- | --- |
| **Screen time** | | | |
| Occasion | Did you use a screen over the past 3 hours? | - Yes - No |  |
| Reason | What were you using the screen(s) for? Check all that apply | - Study - Work - Using social media (e.g., Facebook, Instagram) - Using a messaging service (e.g., WhatsApp) - Watching television or videos (e.g., Netflix, YouTube) - Playing games - Listening to music or podcast - Reading - Other (please describe) |  |
| Duration | How much time did you spend using the screen(s)? | - Less than 15 minutes - 15 to 30 minutes - 31 to 60 minutes - 1 to 2 hours - 2 to 3 hours - More than 3 hours | Adapted from 18-item screen-time questionnaire [42] |
| Screen type | What kind of screen did you watch?  Check all that apply | - Computer/tablet - Smartphone - Smartwatch/activity tracker - Tv/game console - Other (please describe) |  |
| **Behavioural cognitions** | | | |
| Physical activity – intentions | I want to exercise today. | Likert scale   - 1=strongly disagree, 6=strongly agree | Adapted from EMA studies [62, 68] |
| Physical activity – self-efficacy | I am confident I can exercise today. | Likert scale   - 1=not at all confident, 6=extremely confident | Adapted from [48] |
| Physical activity – reflections | Have you done any exercise in today? | - Yes - No | Adapted from EMA studies [62, 68] |

| **Construct** | **Question** | - **Response options** | **Item source** |
| --- | --- | --- | --- |
| Physical activity – barriers | Why not? | - Wasn't planning to - Too tired - Too busy - Bad weather - Risk of injury - No facilities - No one to do it with - Not in the mood - Other (please describe) | Response options taken from [69] |
| Diet – intentions | I want to eat healthy today. | Likert scale   - 1=strongly disagree, 6=strongly agree | Adapted from EMA studies [62, 68] |
| Diet – self-efficacy | I am confident I can avoid unhealthy food and drinks today. | Likert scale   - 1=not at all confident, 6=extremely confident |  |
| Diet – reflections | Did you eat healthy today? | - Yes - No | Adapted from EMA studies [62, 68] |
| Diet – barriers | What prevented you from eating healthy today? Check all that apply | - I was hungry - I felt tired/a lack of energy - I felt bored, stressed, worried, frustrated, lonely, or sad - There was a celebration or others offered/suggested a snack - I saw others eating - I saw an advertisement for delicious food - I saw or smelled delicious food - I noticed food that was good value for money - Other reason | Response options from [70-72] and focus groups in local context [73] |
| **Diet** | | | |
| Eating occasion | Did you eat a meal, snack or drink (other than water) over the past 3 hours? Check all that apply | - Meal - Snacks/desserts - Drink - No | Based on focus group research in local context [73] |

| **Construct** | **Question** | **Response options** | **Item source** |
| --- | --- | --- | --- |
| Food(s) consumed | What foods were part of your meal? Check all that apply | - White rice/noodles/pasta/bread/cereal - Brown or wholegrain rice/noodles/pasta/bread/cereal - Fish or seafood - Chicken - Other meat (e.g., beef, pork, mutton, lamb) - Soy foods (e.g., tofu, tempeh) - Legumes (e.g., nuts, beans, peas, dahl/sambar) - Vegetables - Fruit - Milk, yoghurt or cheese - Any deep-fried food (e.g., puffs, samosas, french fries, fried chicken) - Sweet dessert - Other food (please describe) | Adapted from 37-item Food Frequency Questionnaire developed for local context [44] |
| Food(s) consumed | What snack were you eating? Check all that apply | - Sweet snacks (e.g., cookies, cakes, candy, sweet desserts) - Chips or fried crackers (e.g., potato chips, prawn crackers) - Other deep-fried food (e.g., puffs, samosas, french fries, fried chicken) - Nuts or seeds - Fruits or vegetables - Other snacks (please describe) | Adapted from 37-item Food Frequency Questionnaire developed for local context [44] |
| Drink(s) consumed | What were you drinking? Check all that apply | - Soy or other milk (e.g., almond) - Milk (cow milk) - Malted drink/hot chocolate (e.g., milo) - Fruit juice, 100%, no added sugar - Fruit drinks with added sugar (e.g., ribena) - Other sweetened beverages (e.g., cola, sports drinks, bubble tea, ice tea) - Sugar-free sweetened beverages (e.g., coke zero) - Coffee or tea with sugar (e.g., sugar, condensed milk) - Coffee or tea without sugar - Alcoholic drinks (e.g., beer, wine, spirits) | Adapted from 37-item Food Frequency Questionnaire developed for local context [44] |
| **Construct** | **Question** | - **Response options** | **Item source** |
| Activities while eating | Did you do any of the following while you were eating? Check all that apply | - Study/work - Watch a screen for leisure (e.g., phone, tv) - Play computer games - Reading or writing for leisure - Conversation with others present at the meal - Phone conversation - Walking - Nothing else - Other activity | Adapted from EMA study [74] |
| Food physical environment | Where did you get your meal/snack from? | - Prepared at home - From a food outlet (e.g., Hawker centre, restaurant) - Food delivery (e.g., Grab Food, Deliveroo) - Ready-made meal from a store - Other | Based on mixed methods study of eating out in local context [75] |
| Location | Where did you eat your snack or meal? | - Home - Canteen or dining hall, on-campus - Hawker centre, food court, or coffee shop - Western fast food restaurant - Other restaurant - On-the-go - Other (please describe) | Based on mixed methods study of eating out in local context [75] |
| Snacking reasons | Why did you have a snack? Check all that apply | - It was my regular snack time - I was hungry - I felt tired/a lack of energy - I felt bored, stressed, worried, frustrated, lonely, or sad - For a celebration or others offered/suggested a snack - I saw others eating - I saw an advertisement for delicious food - I saw or smelled delicious food - I noticed food that was good value for money - Other reason | Response options adapted from [71, 72], reasons to snack inventory [70], and focused group discussions [73] |

| **Construct** | **Question** | **Response options** | **Item source** |
| --- | --- | --- | --- |
| Satiety | How full did you feel after eating your meal? | - Still hungry - Neither hungry nor full - Slightly full - Moderately full - Very full - Extremely full | Adapted from [76] |
| **Physical activity** | | | |
| Occasion | Did you do any sport or exercise yesterday? | - Yes - No |  |
| Type | What type of sport or exercise did you do? Check all that apply | - Organised (e.g., instructor-led, classes, team-based) - Self-led - Both |  |
| Purpose | What was the purpose of this sport or exercise? Check all that apply | - Improve health - Improve fitness - Improve appearance (e.g., weight, muscles) - Social - Relaxing, refreshing, or energising - For work - To go somewhere - No purpose - Other (please describe) | Response options taken from [77] |
| Location – physical activity | Where was this? | - Outdoors in parks or green spaces - Outdoors elsewhere - Indoor at home - Indoor elsewhere | Adapted from [78] and used in local context [65, 79] |

| **Construct** | **Question** | **Response options** | **Item source** |
| --- | --- | --- | --- |
| **Binge drinking & smoking** | | | |
| Binge drinking | Did you have 4 (if female) / 5 (if male) or more servings of alcohol at a single drinking session in the past week? | - Yes - No | Used in local cohort studies [38, 40] |
|  | Number of times during the past week you had 4 (if female)/ 5(if male) or more servings of alcohol at a single drinking session in the past week | - Enter number |  |
| Smoking | Did you smoke cigarettes in the past week? | - Yes - No |  |
|  | Number of cigarettes during the past week | - Enter number |  |
| Timings of surveys:   \| Survey 1: 8:30am-9:30am \| \| --- \| \| Survey 2: 11-12pm \| \| Survey 3: 1:30-2:30pm \| \| Survey 4: 4-5pm \| \| Survey 5: 6:30-7:30pm \| \| Survey 6: 9-10pm \| | | | |


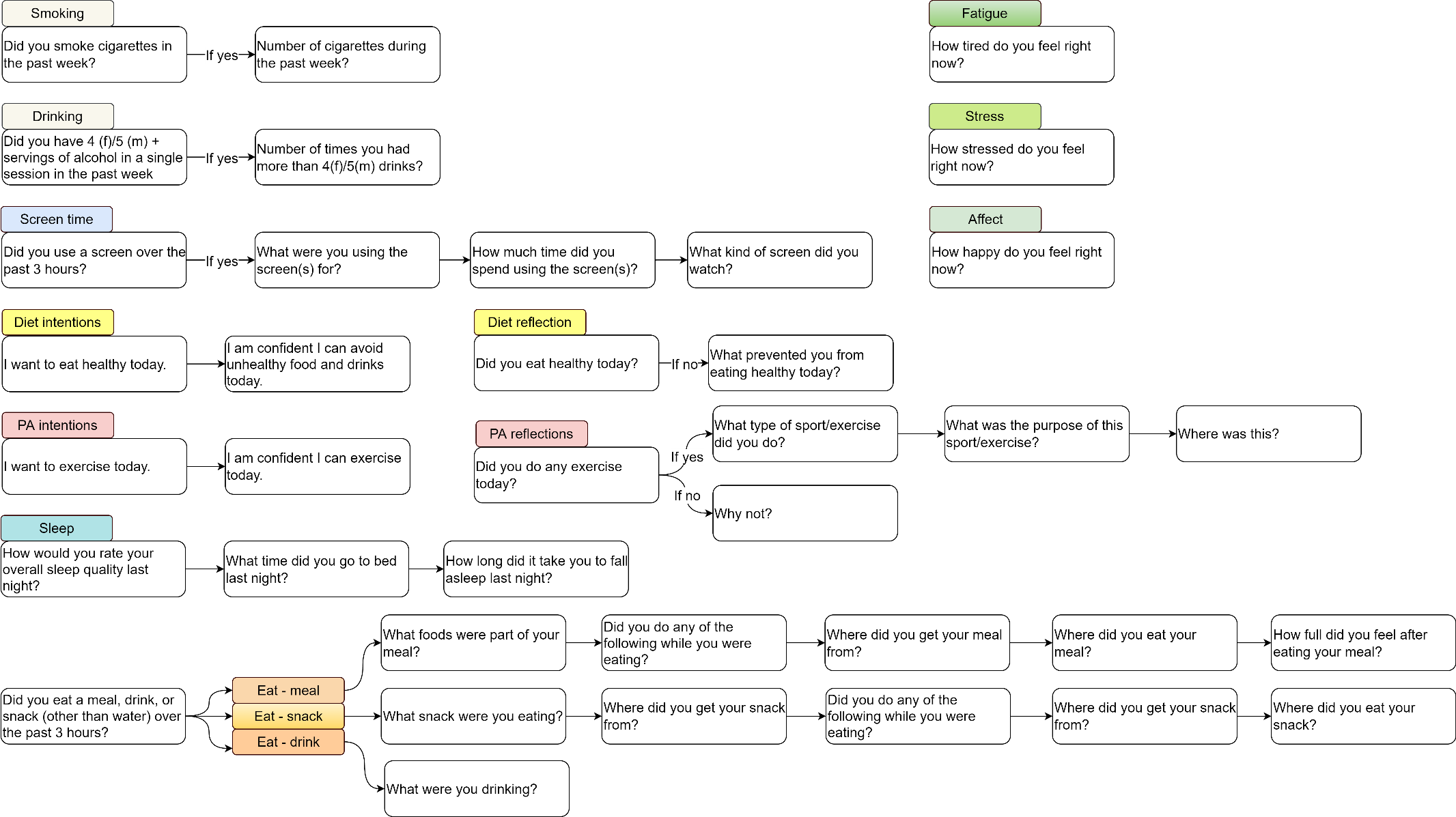


**Supplementary Figure S1.** Tree plot showing an overview of ecological momentary assessment questions across the different question constructs

**Supplementary Figure S2.** Screenshots of hiSG mobile application


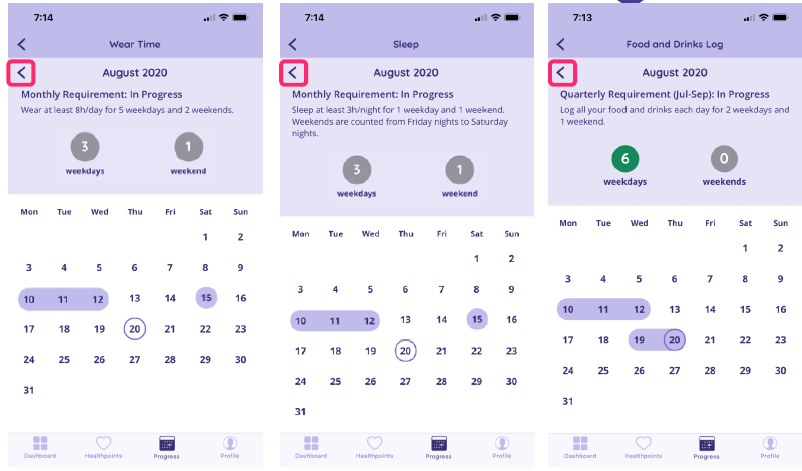


1. Summary of minimum logging requirements (e.g. food logging, wear time and sleep time) for participants to monitor on the hiSG app.


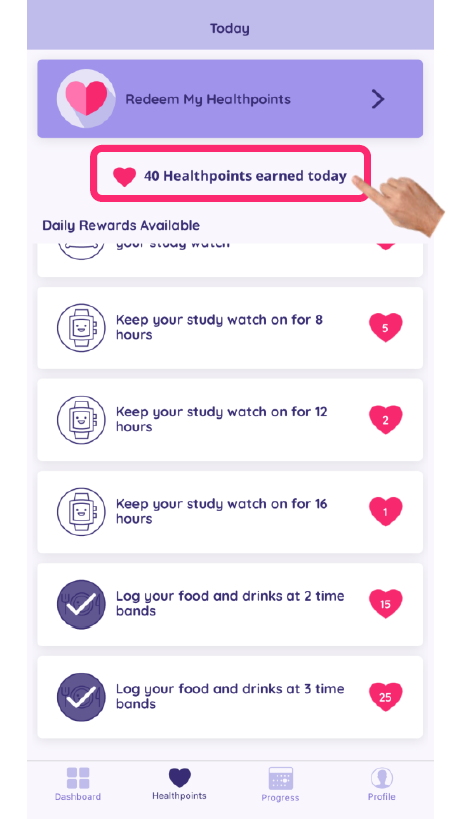


1. Healthpoints tab for participants to claim and redeem their healthpoints for e-vouchers


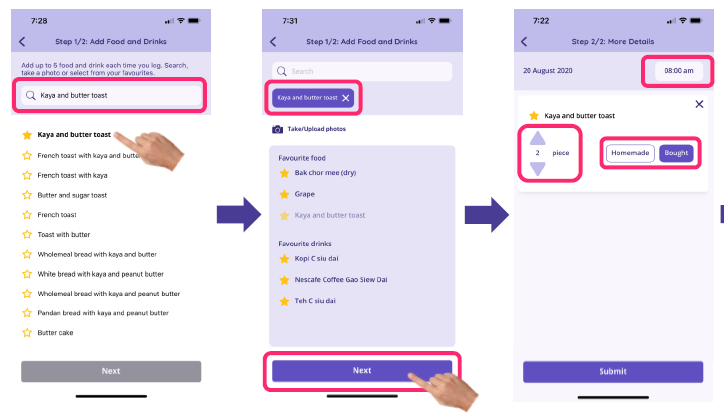


1. Meal logging using the text search function of a food item using the example of “kaya and butter toast”.
